# Predicting the short-term risk of diabetes in HIV-positive patients: the Data Collection on Adverse Events of Anti-HIV Drugs (D:A:D) study

**DOI:** 10.7448/IAS.15.2.17426

**Published:** 2012-10-10

**Authors:** Kathy Petoumenos, Signe W Worm, Eric Fontas, Rainer Weber, Stephane De Wit, Mathias Bruyand, Peter Reiss, Wafaa El-Sadr, Antonella D'Arminio Monforte, Nina Friis-Møller, Jens D Lundgren, Matthew G Law

**Affiliations:** 1 AHOD, The Kirby Institute, University of New South Wales, Sydney, Australia; 2 Copenhagen HIV Programme (CHIP), Faculty of Health Sciences, University of Copenhagen, Copenhagen, Denmark; 3 Nice Cohort, CHU Nice Hopital de l'Archet, Nice, France; 4 SHCS, Division of Infectious Diseases, University Hospital, Zurich, Switzerland; 5 Saint-Pierre Cohort, CHU Saint-Pierre Hospital, Brussels, Belgium; 6 INSERM U897, ISPED, Université Victor Segalen Bordeaux 2, Bordeaux, France; 7 ATHENA, HIV Monitoring Foundation, Academic Medical Center, Amsterdam, the Netherlands; 8 CPPRA Columbia University/Harlem Hospital New York, NY, USA; 9 ICONA Dipartimento di Medicina, Chirurgia e Odontoiatria, Clinica di Malattie Infettive e Tropicali, Azienda Ospedaliera-Polo Universitario San Paolo, Milano, Italy; 10 Department of Infectious Diseases, Copenhagen University Hospital, Copenhagen, Denmark

**Keywords:** HIV, combination antiretroviral treatment, diabetes mellitus, risk equation

## Abstract

**Introduction:**

HIV-positive patients receiving combination antiretroviral therapy (cART) frequently experience metabolic complications such as dyslipidemia and insulin resistance, as well as lipodystrophy, increasing the risk of cardiovascular disease (CVD) and diabetes mellitus (DM). Rates of DM and other glucose-associated disorders among HIV-positive patients have been reported to range between 2 and 14%, and in an ageing HIV-positive population, the prevalence of DM is expected to continue to increase. This study aims to develop a model to predict the short-term (six-month) risk of DM in HIV-positive populations and to compare the existing models developed in the general population.

**Methods:**

All patients recruited to the Data Collection on Adverse events of Anti-HIV Drugs (D:A:D) study with follow-up data, without prior DM, myocardial infarction or other CVD events and with a complete DM risk factor profile were included. Conventional risk factors identified in the general population as well as key HIV-related factors were assessed using Poisson-regression methods. Expected probabilities of DM events were also determined based on the Framingham Offspring Study DM equation. The D:A:D and Framingham equations were then assessed using an internal-external validation process; area under the receiver operating characteristic (AUROC) curve and predicted DM events were determined.

**Results:**

Of 33,308 patients, 16,632 (50%) patients were included, with 376 cases of new onset DM during 89,469 person-years (PY). Factors predictive of DM included higher glucose, body mass index (BMI) and triglyceride levels, and older age. Among HIV-related factors, recent CD4 counts of<200 cells/µL and lipodystrophy were predictive of new onset DM. The mean performance of the D:A:D and Framingham equations yielded AUROC of 0.894 (95% CI: 0.849, 0.940) and 0.877 (95% CI: 0.823, 0.932), respectively. The Framingham equation over-predicted DM events compared to D:A:D for lower glucose and lower triglycerides, and for BMI levels below 25 kg/m^2^.

**Conclusions:**

The D:A:D equation performed well in predicting the short-term onset of DM in the validation dataset and for specific subgroups provided better estimates of DM risk than the Framingham.

## Introduction

HIV-positive patients receiving combination antiretroviral therapy (cART) frequently experience metabolic complications such as dyslipidemia and insulin resistance, as well as lipodystrophy, increasing the risk of cardiovascular disease (CVD) and diabetes mellitus (DM) [1,2]. Rates of DM and other glucose-associated disorders among HIV-positive patients have been reported to range between 2 and 14% [3–6]. In an ageing HIV-positive population who are also on cART, the prevalence of DM is expected to continue to increase. Risk factors for DM in the general population, such as older age, male sex, obesity, lowered high density lipoprotein cholesterol (HDL-C) and raised total cholesterol, have also been found to contribute to the risk of DM in the HIV-positive population [7–10]. Additional factors in HIV-positive patients include lipodystrophy and immunosuppression [8,11], as well as antiretroviral therapy, though the role of antiretroviral therapy remains less clear. An increased risk of DM has been found to be associated with protease inhibitors (PIs) [2,8,12,13] and individual nucleoside reverse transcriptase inhibitors (NRTIs), principally the thymidine analogues [7,14–17], while tenofovir, abacavir and nonnucleoiside reverse–transcriptase (NNRTIs) have not been associated with DM risk [7,8,11,17]. The Data Collection on Adverse Events of Anti-HIV Drugs (D:A:D) study has shown a significant association between new onset DM and exposure to cART, an effect driven mainly by exposure to thymidine analogues [7].

Models for predicting the risk of DM over a five- to ten-year period have previously been developed in the general population [18–20]. These models have included reasonably routinely available patient data such as age, sex, weight, glucose, blood pressure, HDL-C, triglycerides, parental diabetes and receipt of medications. A model based on more complex clinical markers did not predict more accurately than models based on more routine measurements [19]. However, the ability of these DM prediction models to accurately predict the risk of DM in an HIV-positive population is not well known. Furthermore, as diagnosed HIV-positive patients are under routine clinical care, many of these DM risk factors are routinely collected on an ongoing (at least annual) basis [21]; given that current treatment guidelines also recommend metabolic assessment at least annually [21,22], intervention in this population may occur much earlier. In such instances, a model investigating the risk of DM over a shorter period of three to six months (i.e. over the average period between consecutive clinic visits) for patients under active follow-up would more accurately reflect the manner in which HIV-positive patients are followed than one predicting risk five to ten years into the future. A risk equation for CVD in HIV-positive patients, using similar short-term time-varying methodology, has recently been developed by our group [23].

The objective of this current analysis is to develop a model to predict the risk of DM in HIV-positive populations over a short-term period (six months) and to compare this to existing models previously developed in the general population.

## Methods

The D:A:D study is a prospective, multi-cohort observational collaborative study, including 11 previously established cohorts following 33,308 patients at 212 clinics from Europe, Argentina, Australia and the United States. The primary objective of the D:A:D study is to investigate the possible association between cART and the risk of myocardial infarction (MI). The study methodology has been described in detail previously [24]. Briefly, patients were under active follow-up at the individual cohorts at the time of enrolment into the D:A:D study and were included irrespective of whether or how long they were receiving antiretroviral treatment (ART). As part of their routine clinical care data were collected, which include demographic and other prospective patient characteristics such as age, sex, body mass index (BMI) (calculated based on height and weight), CVD, DM, family history of coronary heart disease (CHD), cigarette smoking, blood pressure therapy, DM therapy, lipid lowering and antihypertensive therapy and serum lipid levels (total cholesterol, HDL-C and triglycerides including information on whether values were fasting measures), as well as HIV-related core clinical data including ART medication received, CD4 cell count, viral load and all clinical AIDS diagnoses.

### Definition of DM

DM is a protocol-defined D:A:D secondary endpoint, and all prospective documented cases were verified by the completion of a D:A:D event monitoring case report form. New onset diabetes was defined as either definite, if there was a documented fasting plasma glucose of≥7.0 mmol/L (126 mg/dL) measured on two or more consecutive occasions, or possible, if the patient was recorded as being diabetic with a reported date of onset, and was known to have initiated anti-diabetic therapy. Both definite and possible DM cases were analyzed.

### Statistical methods

The current analyses include all patients recruited to the D:A:D study with follow-up data, without prior DM, MI or other CVD events and with a complete DM risk factor profile. Baseline was defined as the first time point at or after inclusion to the D:A:D study when all DM risk factors were present. Restricting the dataset to patients with a complete risk factor profile allowed for the direct comparison of the D:A:D prediction model with the Framingham model (see “Assessing the performance of the risk equation” below). However, a further analysis was also performed including data on all D:A:D participants, with missing data coded using missing value categories, to assess whether the predictive accuracy of the models altered. Follow-up time was from baseline to the date of new onset DM, death, 1 February 2010 or six months after the patient's last clinic visit, whichever occurred first.

Poisson regression methods were used to determine the factors associated with the short-term (six month) risk of new onset DM in the complete dataset (the development dataset). The predictive model was fitted using time-updated covariates for all laboratory parameters. Although this approach is not generally used when developing prognostic risk equations, it was considered more appropriate in our study for two reasons. First, HIV-positive patients, particularly those on treatment, are routinely seen by their clinicians, at least three or six monthly intervals. Second, over the calendar period the D:A:D study covers, the management of DM and CVD has evolved and improved considerably. A short-term risk equation, therefore, is better placed to accommodate the changes in patient management that have occurred over this period.

Risk factors assessed included the conventional risk factors for DM such as age, sex, fasting and non-fasting glucose (a non-fasting glucose>7.8mmol/L was considered as the equivalent to a fasting value of>5.6 mmol/L) [25], blood pressure (categorized as high: systolic≥130 or diastolic≥85; or receiving blood pressure lowering therapy, and low: systolic<130 and diastolic<85), HDL-C (low:<1.034 mmol/L; median:≥1.034 to<1.550; and high:≥1.550 mmol/L), LDL-C (low:<2.584; median:≥2.584 to<3.359; high:≥3.359 mmol/L), triglycerides (normal:<1.693 mmol/L; borderline to high:≥1.693 to<2.257mmol/L; high:≥2.257 to<5.643; very high:≥5.643) (lipid cut-offs based on ATP III guidelines [26]), BMI per kg/m^2^ and family history of CHD. As a possible alternative to triglycerides and HDL-C as individual measures, the triglyceride to HDL-C ratio was also assessed. However, to avoid over-fitting, only those variables with the best fit in univariate analyses, as determined by the log-likelihood ratio, were assessed in multivariate analyses. In this instance, triglycerides and HDL-C as individual measures performed better in univariate analyses compared with the triglyceride to HDL-C ratio.

In previous analyses smoking was associated with a reduced risk of DM [7,27]. The mechanism for this is unclear, and so to ensure generalizability of results, smoking was not considered as a covariate in these analyses. Subsequent exploratory analyses including smoking did not improve predictions appreciably (data not shown).

The following HIV-related covariates were also assessed: mode of HIV exposure, duration since first HIV-positive test, prior AIDS-defining illness, CD4 cell count, HIV viral load, lipodystrophy and duration of exposure to cART and to each class of drugs. Individual ARTs were also assessed, but limited to those previously determined in the D:A:D study to be significantly predictive of DM (stavudine, zidovudine and didanosine) [7]. Finally, hepatitis C (HCV) and B (HBV) status were also assessed as potential DM risk factors.

All variables were fitted as time-updated covariates (modelling time-updated variables allows for assessment of the short-term risk of new onset DM), while only sex and HIV exposure category were fixed. Lipid parameters, BMI and CD4 cell count were all assessed as continuous as well as categorical covariates; all, apart from BMI, fitted better as categorical rather than continuous. The independent predictors of new onset DM were determined using backward model selection methods. All covariates that were significant at *p*<0.1 in univariate analyses were considered in the multivariate model, and only factors significant at *p*<0.05 in the multivariate model were included in the final predictive model.

### Assessing the performance of the risk equation

The performance of the D:A:D risk equation was assessed using the internal-external cross-validation (IECV) method [28]. Briefly, this approach fits the prognostic model using a leave-one-out cross-validation approach. If there are *k* cohorts, then the model is fitted on a *k*-1 pooled cohort. The performance of the model is then assessed on the cohort that was excluded [29]. Of the 11 cohorts who participate in the D:A:D study, nine cohorts reported all the DM risk factors specified earlier and, therefore, were included in these analyses. Of the nine cohorts, five cohorts reported 20 or more events of DM and were excluded one at a time from the pooled cohort dataset. A further four cohorts each reported fewer than 20 events and were subsequently combined to create the final cohort to be excluded from the pooled dataset. In total, five pooled datasets were created, and the prognostic model was fitted to these datasets one at a time and subsequently assessed for performance on the excluded cohort. Performance was measured as area under the receiver operating characteristic (AUROC) curve, and a weighted average of the five AUROC's was then determined.

The discrimination and accuracy of the equation was also compared with the performance of the Framingham Offspring Study DM equation. We chose the simple clinical model (the model with obesity defined by BMI only) as several of the data for the more complex models are not routinely collected in D:A:D (such as two hour oral glucose tolerance test and fasting insulin levels). Expected eight-year probabilities were determined based on the Framingham algorithm and then converted to a prediction over the shorter D:A:D follow-up period using a linear model. While the Framingham equation might be expected to order patient risk accurately, it might well not be expected to predict the absolute risk accurately. This was previously demonstrated by D'Agostino *et al*. 2001 [30], showing the Framingham equation systematically over-estimating the risk of CHD, although the ordering of the risk was similar [30]. The complete dataset was therefore used to recalibrate the Framingham equation, essentially by increasing the constant terms in the model so that the total number of DM events predicted in the training dataset was equal to the observed number. The Framingham model was also assessed using the aforementioned cross-validation approach, and a weighted average AUROC for this model was also determined.

The AUROC analysis was used to assess the discrimination of the D:A:D study and Framingham risk scores [31], while the accuracy of the risk scores was assessed by comparing the observed versus the predicted number of events for specific subgroups (these were limited to covariates predictive of DM that are in both the D:A:D and Framingham algorithms). The D:A:D risk score was also used to estimate the proportions that were at low (<1%), moderate (1 to 5%), high (5 to 10%) and very high risk (>10%) for DM over a two- and five-year period. The absolute two- and five- year risk was calculated by applying the D:A:D DM risk equation to each individual from the start of their follow-up.

## Results

Of the 33,308 patients followed in the D:A:D study, 16,632 (50%) had a complete DM risk factor profile, with 376 cases of new onset DM during 89,469 PY; the median follow-up was 5.2 years (IQR: 3.0 to 8.1). The incidence of DM in this analyzed population was 4.2 per 1000 PY). Patient characteristics are summarized in Table 1.

**Table 1:** Patient characteristics at baseline

	No diabetes	Diabetes	Total
No. of subjects	16,256	376	16,632
Median follow up (years)	5.3	2.5	5.2
Time at risk (person-years)	88385.3	1083.5	89468.8
	Median (IQR)		
Age (years)	46.1 (40.9 to 52.4)	48.1 (41.8 to 55.5)	46.2 (40.9 to 52.5)
Cumulative cART exposure (years)	2.8 (0.6 to 5.4)	2.9 (1.0 to 4.4)	2.8 (0.6 to 5.3)
Cumulative PI exposure (years)	1.8 (0 to 3.8)	2.0 (0.1 to 3.7)	1.8 (0 to 3.8)
CD4 count (cells/µL)	469 (310 to 665)	432 (271 to 665)	468 (308 to 665)
HIV-RNA (copies/mL)	50 (50 to 2400)	50 (50 to 2240)	50 (50 to 2394)
HDL-C (mmol/L)	1.170 (0.940 to 1.460)	1.037 (0.852 to 1.296)	1.164 (0.931 to 1.457)
Triglycerides (mmol/L)	1.569 (1.027 to 2.490)	2.418 (1.520 to 3.905)	1.580 (1.030 to 2.510)
Trig:HDL-C ratio	1.34 (0.78 to 2.45)	2.41 (1.33 to 4.43)	1.36 (0.78 to 2.49)
Systolic blood pressure	120 (110 to 130)	130 (120 to 140)	120 (110 to 130)
Diastolic blood pressure	80 (70 to 81)	80 (73 to 86)	80 (70 to 82)
BMI (kg/m^2^)	22.9 (20.9 to 25.2)	25.0 (22.8 to 27.9)	23.0 (20.9 to 25.3)
	Number (%)		
Female	27.3	14.9	4498 (27.0)
Current cigarette smoker	51.6	45.0	8151 (51.5)
Ex-smoker	17.9	17.1	2834 (17.9)
Transmission group			
Heterosexual	33.9	35.1	5647 (33.9)
Homosexual	40.9	38.8	6790 (40.8)
Person who injects drugs	19.6	19.7	3261 (19.6)
Other	5.6	6.4	934 (5.6)
Ethnicity			
White	60.6	62.5	10,089 (60.7)
Non-white	7.0	6.6	1157 (7.0)
Other	2.3	1.3	386 (2.3)
Unknown	30.1	29.5	5000 (30.1)
Prior AIDS	24.2	29.0	4036 (24.3)
Hepatitis C positive	27.1	24.9	4156 (25.0)

### The predictive model

In initial univariate analyses, increased time since AIDS diagnosis and smoking were counter intuitively associated with a decreased risk of DM and were excluded from any further consideration. Triglycerides were also found to be much more predictive than the triglyceride:HDL ratio; hence, the ratio was also not considered further.

Factors that were identified as independently predictive of new onset DM included the following: glucose levels more than 5.6 mmol/L for fasting measures or more than 7.8 mmol/L for non-fasting measure (*p*<0.001), increasing BMI (*p*<0.001), high triglycerides (*p*<0.001) and increasing age per five years (*p*<0.001). Among the HIV covariates increasing CD4 category (*p*-trend=0.001) and lipodystrophy (*p*<0.001) were also independently predictive of new onset DM (Table 2). Male gender, increasing total cholesterol, lower HDL-C category, higher systolic or diastolic blood pressure or receiving blood pressure treatment, a prior AIDS diagnosis, any PI use and cumulative use of stavudine were significant in univariate analyses but did not remain significant in multivariate analyses.

**Table 2:** Final predictive models in the complete dataset

		Full model					Model without glucose			
				
	IRR	95%	CI	*p*	*p*	IRR	95%	CI	*p*	*p*
Age per five years	1.16	1.10	1.21	<0.001		1.19	1.13	1.25	<0.001	
Glucose category (mmol/L)										
Less than 7.8 (non-fasting)/5.6 (fasting)										
Greater or equal 7.8 (non-fasting)/5.6 (fasting)	12.89	10.43	15.92	<0.001		–	–	–	–	
Triglycerides (mmol/L)										
Normal (<1.693)										
Borderline to high (≥1.693 to<2.257)	1.87	1.34	2.60	<0.001	<0.001	1.87	1.34	2.62	<0.001	<0.001
High (≥2.257 to <5.643)	2.91	2.23	3.79	<0.001		2.88	2.19	3.79	<0.001	
Very High (>5.643)	5.91	4.23	8.27	<0.001		6.47	4.57	9.16	<0.001	
BMI (per kg/m^2^)	1.10	1.08	1.13	<0.001		1.13	1.10	1.15	<0.001	
CD4<200 cells/µL										
CD4≥200 to<350 cells/µL	0.52	0.36	0.77	0.001	<0.001	0.49	0.33	0.72	<0.001	<0.001
CD4≥350 cells/µL	0.51	0.37	0.69	<0.001		0.48	0.35	0.66	<0.001	
Lipodystrophy	1.27	1.02	1.56	0.029						
HDL-C (mmol/L)										
Low (<1.034)										
Median (≥1.034 to<1.550)	–	–	–	–		0.77	0.62	0.96	0.021	0.017
High (>1.550)	–	–	–	–		0.73	0.51	1.03	0.071	
High BP (SYS≥130 or DIA≥85, or on BP treatment)	–	–	–	–		1.37	1.09	1.72	0.007	

*p* values reported for test of homogeneity in nominal covariates and test for trend for ordinal covariates. Patients with data no recorded were not included when testing trend. IRR, Incidence rate ratio.

### Performance of the D:A:D and Framingham risk equations

From the IECV process, the D:A:D risk equation yielded a weighted average AUROC of 0.894 (95% CI:0.849, 0.940), while the recalibrated Framingham algorithm yielded a weighted average AUROC of 0.877 (95% CI: 0.823, 0.932).

Observed and predicted numbers of new onset DM events for the D:A:D and the recalibrated Framingham risk equations by key patient subgroups (factors common in both the Framingham and D:A:D risk equations) are summarized in Table 3. The Framingham equation predicted 258.5 new onset DM events in the complete dataset and was recalibrated to predict the observed 376. As might be expected, the D:A:D equation fitted marginal subgroup totals better than the recalibrated Framingham equation. The Framingham algorithm over-predicted DM events compared to the D:A:D model for those with lower glucose (219 and 146 events, respectively, compared with the observed 141 events), lower triglycerides (116 and 87 events, respectively, versus an observed 84 events), and slightly lower for those with BMI levels between 26 and 29 (80 and 95 events compared with an observed 107 events) and BMI above 30 kg/m^2^ (52 and 60 events compared with an observed 60 events).

**Table 3:** Observed versus predicted events

		Predicted	
		
	Observed	D:A:D	Framingham recalibrated
Glucose category			
Less than 7.8 (non-fasting)/5.6 (fasting)	141	146.4	219.1
Greater or equal 7.8 (non-fasting /5.6 (fasting)	235	229.6	156.9
Triglycerides (mmol/L)			
Normal (<1.693)	84	86.5	116.5
Borderline to high (≥1.693 to<2.257)	60	61.1	79.4
High (≥5.257 to<5.643)	171	169.8	152.4
Very High (>5.643)	61	58.5	27.7
BMI (kg/m^2^)			
BMI<18 (underweight)	10	5.5	9.9
BMI≥18 to<26 (normal weight)	199	217.8	234.4
BMI≥26 to<30 (overweight)	107	93.1	80.0
BMI>30 (obese)	60	59.6	51.8

The absolute two- and five-year risk is shown in Table 4. Two and six percent of the study population was estimated to be at high risk, and less than 1% and 4% at very high risk, of developing DM over two and five years, respectively. These proportions were considerably lower among females compared to males, and among younger (<40 years) compared to older individuals.

**Table 4:** DM two- and five-year risk stratification

	Low	Moderate	High	Very High
	
	<1%	1 to 5%	5 to 10%	>10%
Two-year risk				
% of population	79.13	17.78	2.25	0.84
% of men	75.78	20.40	2.80	1.02
% of women	88.17	10.72	0.76	0.36
% of younger individuals (<40 yr)	88.63	10.46	0.80	0.12
% of older individuals (40+ yr)	70.76	24.23	3.53	1.48
Five-year risk				
% of population	54.37	35.25	6.13	4.25
% of men	48.13	39.54	7.21	5.12
% of women	71.19	23.68	3.22	1.91
% of younger individuals (<40 yr)	69.63	25.90	3.07	1.40
% of older individuals (40+ yr)	40.91	43.49	8.83	6.76

We also developed a DM prediction model excluding glucose, the key predictor in both the D:A:D and Framingham models, essentially to identify HIV-positive patients who might be at raised risk of DM and who subsequently should have a fasting glucose assessment to allow more accurate prediction. This model included many of the same covariates identified in the full model previously discussed, such as age, BMI, triglycerides and CD4 cell count. In addition, this model also included HDL-C and blood pressure. As with the model including glucose, male gender, increasing total cholesterol, prior AIDS diagnosis, any PI use and cumulative D4T use were not included in the final model. The mean weighted average AUROC for this model was 0.756 (95% CI: 0.737 to 0.775).

Finally, in the sensitivity analysis, where all D:A:D patients with missing data were included using missing data categories, the weighted average AUROC for the D:A:D equation was 0.847 (95% CI: 0.780 to 0.914).

## Discussion

We developed a short-term risk equation for the prediction of new onset DM in a cohort of HIV-positive individuals. Our risk equation included traditional DM risk factors age, impaired glucose, and triglycerides as well as HIV-related factors, HIV immunosuppression and the presence of lipodystrophy. To our knowledge, this is the first diabetes risk equation using routinely collected clinical data developed specifically for HIV-positive patients. This risk equation was found to be relatively robust with good discrimination when assessed by IECV methods and, as might be expected, was also shown to have better overall performance and discrimination to the Framingham risk equation.

Among the conventional risk factors, one notable exception in the D:A:D risk equation is male gender. Despite gender being identified as an independent predictor of new onset DM in a previous D:A:D analysis [7], it is not included in the final D:A:D risk equation. The discrepancy between our current analyses and prior D:A:D findings may largely be explained by the smaller overall population included in this analysis, almost half of that in previous D:A:D study. Furthermore, the previous D:A:D analysis did not adjust for lipids. Data from the Swiss HIV Cohort Study (SHCS) also reported male gender as significant predictor of new onset DM, yet similar to the previous D:A:D analyses, it did not adjust for lipids [32]. Both the D:A:D study and the SHCS reported risk ratio of 1.6 and 1.7, somewhat larger than our current analyses of 1.1.

Among the HIV-related factors previously reported to be significantly predictive of new onset DM not included in our final risk equation model is antiretroviral treatment. Cumulative stavudine and any PI use were significant in univariate analyses but dropped out of the final model. Several studies, including D:A:D previously, have reported significant increased risk of DM with ART use. Previously shown in the D:A:D study was an increased risk of DM with NRTI use, specifically stavudine, zidovudine and didanosine, and a decreased risk with ritonavir and nevirapine [7]. However, data from the MACS cohort reported an increased risk of DM with ritonavir and saquinvar and a decreased risk with indinavir and nelfinavir [12]. Given the ongoing debate and inconsistency in associations of ART use and DM onset across different populations, excluding ART treatment from a risk equation for HIV-positive patients may in fact improve the robustness of the equation and allows the current equation to be used into the future where patterns of ART use will continue to evolve.

Hepatitis C co-infection has also been shown in some studies [12,33], but not all studies [8], to be associated with DM. In our current analysis, we did not find HCV to be a predictor of new onset DM. Studies that have reported an association also report substantially greater incidence of DM. In the Multi-Centre AIDS Cohort Study (MACS), DM incidence ranged from 47 per 1000 PY for those receiving cART and 17 per 1000 PY in those not on cART [12]. Rates in our study were much lower than MACS, 4.2 per 1000 PY similar to that reported in the SHCS, who also did not find an association between HCV and DM [8].

### Performance

The D:A:D risk equation performed marginally better than the Framingham equation, both overall, in terms of discriminating between key subgroups such as those defined by glucose, triglyceride levels and, to a lesser extent, BMI. Nevertheless, the recalibrated Framingham also performed well. The fact that the D:A:D analyses identified several of the same predictors as the Framingham model is reassuring, suggesting that key parameters, such as glucose and triglycerides, may be interpreted qualitatively similarly in HIV-positive populations as negative populations. However, we were unable to compare the D:A:D model with other established DM risk equations as the necessary data are not routinely collected in the D:A:D study [18,20,34] – thus, it remains uncertain how these models would perform in HIV-positive populations compared with either the Framingham or the D:A:D equation.

There are some limitations to our analyses. First, we included non-fasting as well as fasting glucose measures, although accepted cut-offs for non-fasting glucose measures were applied [25]. Second, family history of CVD was used as a surrogate for family history of DM as this information is not available in the D:A:D study. Third, we do not have data on other factors previously identified as predictive of DM, including waist circumference. Race has also been associated with DM in both HIV and non-HIV studies [7,18] and has been included in at least one DM risk model [18]. We were unable to assess race in the present model as a substantial proportion of the events occurred in the unknown race category, as many of the collaborating cohorts do not routinely report race. Exclusion of race in the D:A:D model, however, makes our risk score more generalizable across HIV cohorts from various regions and ethnic backgrounds. Finally, in order to compare the D:A:D prediction model with the Framingham model, we included only patients who had data for all the pre-defined risk factors. Consequently, half of the D:A:D cohort were excluded from the main analyses. If these patients had differed considerably from the patients included in the analyses, the generalizability of the prediction equation may have been affected. However, the sensitivity analysis which included the entire D:A:D cohort yielded a very similar AUROC to the main analysis, demonstrating the robustness of the equation.

A key strength of our equation is that it predicts the short-term risk of DM, including variables that vary over time. First, this short-term nature reflects the way HIV-positive patients are managed, with regular clinic visits. Second, as early intervention is possible, changes in management fit better with this equation. Prediction of DM is also important in HIV-positive cohorts because, as well as being a notable condition in its own right, DM is also associated with the development of more serious outcomes, in particular CVD [23,35].

### Application

Prevention and management of DM is becoming increasingly important as HIV-positive people in the era of effective ART are living longer, and the prevalence of DM is expected to continue to increase. The D:A:D DM risk equation may be used in a clinical setting by doctors treating HIV-positive patients. The automatic calculation of the patient's short-term risk for DM would be beneficial in identifying more efficiently patients at risk of developing DM and subsequently other CVDs. Current guidelines recommend metabolic assessment among HIV patients when commencing ART, at time of switching therapy, and at three to six months after commencing or switching ART and once yearly during stable therapy [21,22]. Assessment of the short-term risk of new onset DM, therefore, may also occur with very little additional inconvenience to the patient. The model without glucose may also be used to screen patients and identify those who are at very low risk of DM and therefore do not require more frequent screening for glucose. Both prediction models (with and without glucose) will be made publicly available on the D:A:D website (http://www.cphiv.dk/) and can be used for individual patients. While predictions are only a guide and should not be over interpreted, they do allow for patients at high risk to be identified, and appropriate interventions may then be applied. Calculating an individuals predicted risk is desribed in the appendix.

## Appendix

The risk of DM is estimated as:

1 – exp ^(−H**t*)^; where

$$\text{H}=\text{ex}{\text{p}}^{{\text{β}}_{\text{0}}+{\text{β}}_{\text{1}}{\text{x}}_{\text{1}}+{\text{β}}_{\text{2}}{\text{x}}_{\text{2}}+{\text{β}}_{\text{3}}{\text{x}}_{\text{3}}+{\text{β}}_{\text{4}}{\text{x}}_{\text{4}}+{\text{β}}_{\text{5}}{\text{x}}_{\text{5}}+{\text{β}}_{\text{6}}{\text{x}}_{\text{6}}+{\text{β}}_{\text{7}}{\text{x}}_{\text{7}}+{\text{β}}_{\text{8}}{\text{x}}_{\text{8}}+{\text{β}}_{\text{9}}{\text{x}}_{\text{9}}}$$

β_0_=−10.176

β_1_x_1_=0.144 per 5 years of age (e.g. if age=40 then β_1_x_1_=((40/5)*0.144)

β_2_x_2_=2.556 (x_2_=1 if glucose >7.8 non-fasting/5.6 fasting, x_2_=0 if not)

β_3_x_3_=0.623 (x_3_=1 if TRIG≥1.693 to<2.257 mmol/L, x_3_=0 if not)

β_4_x_4_=1.069 (x_4_=1 if TRIG≥2.257 to<5.643 mmol/L, x_4_=0 if not)

β_5_x_5_=1.777 (x_5_=1 if TRIG>5.643 mmol/L, x_5_=0 if not)

β_6_x_6_=0.098 per unit increase of BMI (e.g. if BMI=25 then β_6_x_6_=(25*0.165)

β_7_x_7_=−0.646 (x_7_=1 if CD≥4200 to <350 cells/µL, x_7_=0 if not)

β_8_x_8_=−0.677 (x_8_=1 if CD4≥350 cells/µL, x_8_=0 if not)

β_9_x_9_=0.236 (x_9_=1 if lipodystrophy present, x_9_=0 if not)

Data in the D:A:D study are set up in monthly time units (0.085 years); the above equation therefore produces a monthly probability of developing DM. A reasonably good approximation for calculating the estimated probability of DM over longer time periods, *t*, is to multiply “H” by *t* years, and use age+*t*/2 in the equation. More exact computation will be available through a calculator on the D:A:D website (http://www.cphiv.dk/).

**Worked example:**

Consider an individual is male, 48.7 years of age, has fasting glucose of >5.6 mmol/L, BMI of 27.5, triglyceride measure of 2.65 mmol/L, CD4 cell count of 550 cell/µl, and with lipodystrophy

To calculate the six month estimated risk of DM, we first calculate:

β_1_x_1_=calculated as 0.144*((48.7+0.25)/5), β_2_x_2_=2.556,

β_3_x_3_=0, β_4_x_4_=1.069, β_5_x_5_=0, β_6_x_6_=0.098*27.5, β_7_x_7_=0,

β_8_x_8_=−0.677, β_9_x_9_=0.236.

Then H**t*=0.5*0.0556=0.0278

Therefore, the converted six month hazard (i.e. six month risk of DM)=2.74 per 1000 PY.
